# Delirium in neurosurgery: a systematic review and meta-analysis

**DOI:** 10.1007/s10143-021-01619-w

**Published:** 2021-08-16

**Authors:** P. R. Kappen, E. Kakar, C. M. F. Dirven, M. van der Jagt, M. Klimek, R. J. Osse, A. P. J. E. Vincent

**Affiliations:** 1 Department of Neurosurgery, Erasmus MC, University Medical Center Rotterdam, Rotterdam, Netherlands; 2 Department of Neuroscience, Erasmus MC, University Medical Center Rotterdam, Rotterdam, Netherlands; 3 Department of Intensive Care Adults, Erasmus MC, University Medical Center Rotterdam, Rotterdam, Netherlands; 4 Department of Anesthesiology, Erasmus MC, University Medical Center Rotterdam, Rotterdam, Netherlands; 5 Department of Psychiatry, Erasmus MC, University Medical Center Rotterdam, Rotterdam, Netherlands

**Keywords:** Neurosurgery, Delirium, Screening tools, Incidence rates

## Abstract

Delirium is a frequent occurring complication in surgical patients. Nevertheless, a scientific work-up of the clinical relevance of delirium after intracranial surgery is lacking. We conducted a systematic review (CRD42020166656) to evaluate the current diagnostic work-up, incidence, risk factors and health outcomes of delirium in this population. Five databases (Embase, Medline, Web of Science, PsycINFO, Cochrane Central) were searched from inception through March 31st, 2021. Twenty-four studies (5589 patients) were included for qualitative analysis and twenty-one studies for quantitative analysis (5083 patients). Validated delirium screening tools were used in 70% of the studies, consisting of the Confusion Assessment Method (intensive care unit) (45%), Delirium Observation Screening Scale (5%), Intensive Care Delirium Screening Checklist (10%), Neelon and Champagne Confusion Scale (5%) and Nursing Delirium Screening Scale (5%). Incidence of post-operative delirium after intracranial surgery was 19%, ranging from 12 to 26% caused by variation in clinical features and delirium assessment methods. Meta-regression for age and gender did not show a correlation with delirium. We present an overview of risk factors and health outcomes associated with the onset of delirium. Our review highlights the need of future research on delirium in neurosurgery, which should focus on optimizing diagnosis and assessing prognostic significance and management.

**Supplementary Information:**

The online version contains supplementary material available at 10.1007/s10143-021-01619-w.

## Introduction

Delirium is characterized by a temporary decline in the patient’s mental status affecting attention, awareness, cognition, language and/or visuospatial ability, [1] caused by dysregulation of neuronal activity [2]. Intracranial surgery evokes a parenchymal inflammatory reaction resulting in oxidative stress, which is subsequently aggravated by impaired oxygenation of the surrounding tissue due to the formation of oedema. Hypotheses describing the pathophysiology of delirium include neuro-inflammatory and oxidative reactions within the brain. Considering this, neurosurgical patients are vulnerable to delirium [2].

Unfortunately, delirium in the neurosurgical population has been under-investigated. This may be explained by the lack of consensus on definition and challenge with respect to its diagnosis [3–5]. Therefore, reported incidences vary, especially in case of hypoactive delirium 6]. Delirium is considered a severe complication in other populations, being a traumatic experience for patients and contributing to prolonged hospital stay, higher risk for re-operation, mortality and cognitive decline [7–10]. These consequences of delirium led to increased research on delirium, including in the neurosurgical population [5, 7, 9, 11].

In order to assess the current knowledge regarding the diagnostic work-up, incidence, risk factors and health outcomes associated with post-operative delirium in hospitalized neurosurgical patients with primary brain pathologies, we conducted a systematic review and meta-analysis.

## Methods

### Protocol and registration

This study follows the guideline from Meta-Analysis of Observational Studies in Epidemiology (MOOSE) [12] and is registered in the PROSPERO database (CRD42020166656).

### Search strategy

The literature search was conducted with a dedicated biomedical information specialist. The electronic databases Embase, Medline, Web of Science, PsycINFO and Cochrane Central were searched from date of inception through March 31st, 2021 (Appendix 1).

### Study selection and eligibility criteria

Two reviewers (PK/EK) independently screened the title/abstract according to a standardized protocol [13]. Of note, we have decided to only include patients that underwent intracranial surgery (with and/or without requirement of bone flap removal) to assess delirium as a post-operative complication to improve the uniformity of the study population, which is a minor adaptation from the original protocol as registered in PROSPERO. Prospective, retrospective cohort studies and randomized controlled trials (RCTs) were included. Exclusion criteria were extra-cranial neurosurgical procedures, case series with a sample size of < 10 patients and English full text not available. Full-text screening required a clear number of patients that underwent intracranial surgery and reproducible diagnosis of delirium, with or without the use of a validated tool (e.g. just mentioning delirium without detail on diagnostic assessment would lead to exclusion).

### Data extraction and data items

Data, including author name, year of publication, study design, baseline characteristics, method of delirium assessment, cohort size (including incidence of delirium), risk factors and health outcomes, were extracted independently by the same two reviewers (PK/EK). The primary outcome was method of delirium assessment (validated vs non-validated tools, daily frequency and follow-up). Secondary outcomes included the incidence, risk factors and delirium-related health outcomes associated with post-operative delirium. In case of a RCT, only data of the control group were used. Risk factors and health-related outcomes were evaluated in studies using validated delirium assessment tools (i.e. delirium assessment tools validated within any hospital-based population) [14].

### Risk of bias assessment

The same two reviewers (PK/EK) independently evaluated the risk of bias. For RCTs, the Cochrane Collaboration’s risk of bias tool was used [15]. Non-randomized trials were evaluated using the Newcastle–Ottawa Scale (NOS). The NOS’s was adapted, after individually appraising the first five articles, due to its poor inter-observer reliability (Cohen’s kappa = 0.29) (Appendix 2) [16, 17]. The grade of certainty across studies was assessed using the Grading of Recommendations Assessment, Development and Evaluation (GRADE) approach.

### Statistical analysis

Descriptive statistics were presented as counts (*n*, %) and means (standard deviation (SD)). Medians, in case of skewed variables, were used as approximation of the mean. Interquartile ranges (IQR) were divided by 1.35 as approximation of the SD. Reported confidence intervals (95%CI) were used to approximate the SD (= ((CI upper limit–CI lower limit)/3.92)*(root square of the cohort size)). The widths of reported ranges were divided by four as approximation of the SD.

Meta-analysis of proportions was performed using the random effects model with the restricted maximum likelihood method, since within and between-study variance was expected. Proportions were defined as the fraction of patients with delirium. Before pooling, all data were transformed, using the Freeman–Tukey double arcsine transformation, to correct for extreme proportions (e.g. < 0.2 and > 0.8) and small sample sizes [18]. Heterogeneity was assessed using the *I*^2^ statistics. Outliers were identified by screening for externally studentized residuals of > 3 and excluded if the outlier caused significant changes in the meta-analysis [19]. Subgroup analysis was performed based on clinical features and delirium diagnosis method. Delirium-associated significant multivariate risk factors and health outcomes were presented as odds ratio’s (ORs) with CIs. Meta-regression was performed for risk factors if ≥ 8 studies were available. We did qualitative analysis for delirium-related risk factors and health outcomes, when studies reported multivariable associations. Data were analysed using R version 4.0.0, and a *p* value of < 0.05 was considered statistically significant.

## Results

### Systematic search

Our search, last update conducted on March 31st, 2021, yielded 6974 studies (Appendix 3). A total of 4290 studies were screened on the title/abstract. Eventually, 47 studies were assessed full text, of which 27 excluded: delirium diagnosis not reproducible (*n*_s_ (number of studies) = 9), [20–28] full text not found (*n*_s_ = 3), [29–31] duplicate (*n*_s_ = 3), [18, 32] paediatric patients (*n*_s_ = 1) [33], overlapping populations (*n*_s_ = 3), [4, 34, 35] no delirium assessment (*n*_s_ = 1), [36] no original data (*n*_s_ = 2) [37, 38] and an unclear number of patients undergoing intracranial surgery (*n*_s_ = 5) [39–43]. Finally, 20 paper were included in the qualitative analysis and 18 papers in the quantitative analysis (*n*_p_ (number of patients) = 5083).

### Study and patient characteristics

Table 1 describes the study and patient characteristics. Two RCTs, seven prospective and eleven retrospective cohort studies were included. Disease type for patients undergoing intracranial surgery were categorized in mixed (33.9%, *n*_p_ = 1478), [4, 10, 21, 32, 39, 41–48] functional neurosurgery (26.8%, *n*_p_ = 552), [11, 46, 49–51] neurovascular (10.5%, *n*_p_ = 145), [52–54] neuro-oncology (18.4%, *n*_p_ = 1969) [5, 7, 55], traumatic brain injury (TBI) (4.3%, *n*_p_ = 27) [56, 57] and microvascular decompression (MVD) (6.2%, *n*_p_ = 912) [9]. The mixed group included neurovascular, neuro-oncologic, TBI or hydrocephalus operations and functional neurosurgery (solely deep brain stimulation (DBS) in patients with Parkinson’s disease). Twelve studies assessed delirium in neurosurgical patients in the nursing ward, [7, 9–11, 23, 39, 41–43, 45, 46, 49–52, 56, 57] six studies in the ICU [4, 32, 46–48, 53, 54] and two studies in both [44, 55]. Six studies did not specify the number of patients undergoing craniotomy (i.e. requiring removal) [10, 21, 39, 44, 46–48, 52, 54, 56, 57]. Six studies did not report age, and seven studies did not report gender within the intracranial operated cohort [45, 47–49, 52–54]. Pooled age in years (mean/SD, *n*_s_ = 14) [4, 5, 7, 9–11, 32, 44, 46, 49–51, 55, 56] and percentage of males (*n*_s_ = 13) [5, 7, 9–11, 32, 44, 46, 50, 51, 55, 56] of the remaining studies were 60.32% (4.47) and 49.6%, respectively.

**Table 1:** Baseline table

**Author**	**Study design**	**Context**	**Type of disease**^**1**^	**Cohort size intracranial**^**2**^	**Cohort size craniotomy**^**3**^	**Age**^**4**^	**Gender**^**5**^
Budenas, 2018	Prospective cohort	Ward	Neuro-oncology	522	446	57.2/15.0	63.3
Carlson, 2013	Retrospective cohort	Ward	Functional	59	0	65.0/8.7	NR
Chen, 2020	Retrospective cohort	Ward and ICU	Neuro-oncology	893	893	47.8/14.4	55.5
Flanigan, 2017	Retrospective cohort	Ward	Neuro-oncology	554	500	60.8/12.8	41.0
Greenberg, 2017	RCT	ICU	Mixed	65	65	56.0/15.0	55.4
Harasawa, 2014	Prospective cohort	Ward	Neurovascular	98	98	NR	NR
He, 2019	Retrospective cohort	Ward	MVD	912	912	59.6/10.6	61.2
Hosoya, 2018	Retrospective cohort	ICU	Neurovascular	32	13	NR	NR
Lange, 2015	Retrospective cohort	Ward	Functional	38	0	64.1/17.8	34.2
Matano, 2017	Prospective cohort	Ward	Mixed	65	NR	64.1/18.75	45.5
Mokhtari, 2020	RCT	ICU	Mixed	16	NR	NR	NR
Morshed, 2019	Retrospective cohort	Ward and ICU	Mixed	235	NR	52.6/15.3	50.6
Ogasawara, 2000	Prospective cohort	Ward	TBI	27	NR	80.4/3.8	25.9
Oh, 2008	Retrospective cohort	Ward	Mixed	75	0	NR	NR
Tanaka, 2018	Retrospective cohort	Ward	Functional	61	0	65.6/9.2	55.7
Wang, 2020A	Prospective cohort	ICU	Mixed	800	0	48.0/12.5	59.0
Wang, 2017	Prospective cohort	ICU	Neurovascular	47	40	NR	NR
Wang, 2019	Retrospective cohort	Ward	Functional	165	NR	60.6/9.21	48.0
Wang, 2020B	Prospective cohort	ICU	Mixed	238	NR	NR	NR
Zhan, 2020	Retrospective cohort	Ward	Functional	229	0	62.71/6.41	47.6
Overall				5131	2967	60.32/4.47	49.6

^1^Patients operated for either neurovascular, neuro-oncologic, traumatic brain or hydrocephalus. ^2^Sample size of patients having undergone intracranial surgery (including biopsy, ventricular drainage). ^3^Among which, the number of patients undergoing intracranial surgery requiring bone flap removal. ^4^Age, mean and standard deviation. ^5^Gender, percentage female; *MVD*, microvascular decompression; *RCT*, randomized controlled trial; *TBI*, traumatic brain injury; *NR*, not reported

### Delirium diagnosis

Fourteen (70.0%) studies used validated delirium assessment tools (Table 2). One (5.0%) study confirmed delirium, in patients using Delirium Observation Screening Scale (DOS) scores > 2, in combination with the Diagnostic and Statistical Manual of Mental Disorders (DSM) criteria [9, 39]. Most studies (*n*_s_ = 9 (45.0%)) used the Confusion Assessment Method (CAM) or the modified version for the intensive care unit (CAM-ICU) as a diagnostic or screening tool. The CAM(-ICU) in all studies was defined as positive for delirium when three out of four items were scored positive [4, 7, 32, 42, 45–48, 51, 53, 55]. Two (10.0%) studies assessed delirium using the Intensive Care Delirium Screening Checklist (ICDSC) [10, 39, 41, 54]. One (5.0%) study, assessed delirium using the Neelon and Champagne (NEECHAM) Confusion Scale, defined delirium as positive in case of once a score of < 24 or a score of < 27 for 2 consecutive days [52]. One (5.0%) study used the Nursing Delirium Screening Scale (Nu-DESC), as an alternative for the CAM-ICU, and considered delirium positive in case of a score ≥ 2 [44].

**Table 2:** Delirium diagnosis

Author	Definition delirium diagnosis	Instrument	Validated^1^		Period delirium screening^2^	Frequency screening^3^
Budenas, 2018	One positive CAM-ICU: 3 out of 4 positive features 4 positive features	CAM-ICU	Yes	Day 2–7		NR
Carlson, 2013	Occurrences of any event of hallucinations, delusions or disorientation to circumstance, even if apparently benign	Own definition	No	Until discharge		NR
Chen, 2020	Positive CAM-ICU	CAM-ICU	Yes	Within 72 h		Three times
Flanigan, 2017	Acute state of confusion and disorientation with changes in arousal/attention. Confusion without changes in arousal was considered mutually exclusive with delirium	Own definition	No	Within 72 h		NR
Greenberg, 2017	Positive CAM-ICU	CAM-ICU	Yes	Within 24 h		Three times
Harasawa, 2014	Neecham (0–30) with cut-off 24 or less OR 27 2 consecutive days	NEECHAM	Yes	Day 1–3		NR
He, 2019	DOS (three or greater) confirmed with DSM-5 by psychiatrist	DOS	Yes	Day 2–5		NR
Hosoya, 2018	ICDSC 4 or higher	ICDSC	Yes	Until discharge		NR
Lange, 2015	Altered mental state of reduced cooperation due to fear, psycho-motor agitation and impaired or lost orientation	Own definition	No	Day 1–30		NR
Matano, 2017	ICDSC 4 or higher	ICDSC	Yes	Day 1–7		Two times
Mokhtari, 2020	Positive CAM-ICU	CAM-ICU	Yes	Day 1–7		Two times
Morshed, 2019	Either CAM-ICU (1 and 2 and 3 and/or 4) or Nu-DESC (2 or higher) once positive	CAM-ICU/Nu-DESC	Yes	Until discharge		NR
Ogasawara, 2000	Vivid hallucination, delusion, extreme agitation, irritability and signs of over activity in the autonomic nervous system	Own definition	No	NR		NR
Oh, 2008	Positive for delirium when MMSE less than 23 OR positive CAM-ICU (1 and 2 and 3 and/of 4)	MMSE/CAM-ICU	No	Day 1–3		NR
Tanaka, 2018	Any event involving hallucinations, delusions or disorientation to circumstance including any attempt to remove the urinary catheter or peripheral venous catheter	Own definition	No	Day 1–14		NR
Wang, 2020A	Positive CAM-ICU (either 1 and 2 with 3 and/or 4)	CAM-ICU	Yes	Day 1–3		One time
Wang, 2017	Positive CAM-ICU (either 1 and 2 with 3 and/or 4)	CAM-ICU	Yes	Until discharge		Two times
Wang, 2019	Positive CAM-ICU (either 1 and 2 with 3 and/or 4)	CAM-ICU	Yes	Day 1		NR
Wang, 2020B	According to guidelines: ICU guidelines	CAM-ICU	Yes	Until discharge		Two times
Zhan, 2020	Positive CAM-ICU (either 1 and 2 with 3 and/or 4)	CAM-ICU	Yes	Day 1		One time

^1^Validated tools for delirium screening. ^2^Follow-up duration for delirium screening. ^3^Daily frequency of delirium screening. *NR*; not reported

Six (30.0%) studies used non-validated, but reproducible, screening tools for delirium. One study, assessing delirium with either the Mini-Mental State Examination (MMSE) or CAM-ICU, did not separately report values for the CAM-ICU and was therefore considered non-validated [45]. The remaining studies predefined their tools based on own defined criteria [11, 21, 49, 50, 56].

A follow-up period for delirium assessment was reported in all but one study [56], which varied from 24 h to 30 days. Frequency of daily delirium screening was specified in eight (40.0%) studies: three times per day (*n*_s_ = 2), [32, 39, 43, 55] twice per day (*n*_s_ = 4) [10, 46, 48, 53] and once per day (*n*_s_ = 2) [4, 46, 51, 57].

### Incidence of delirium

**Fig. 1:**
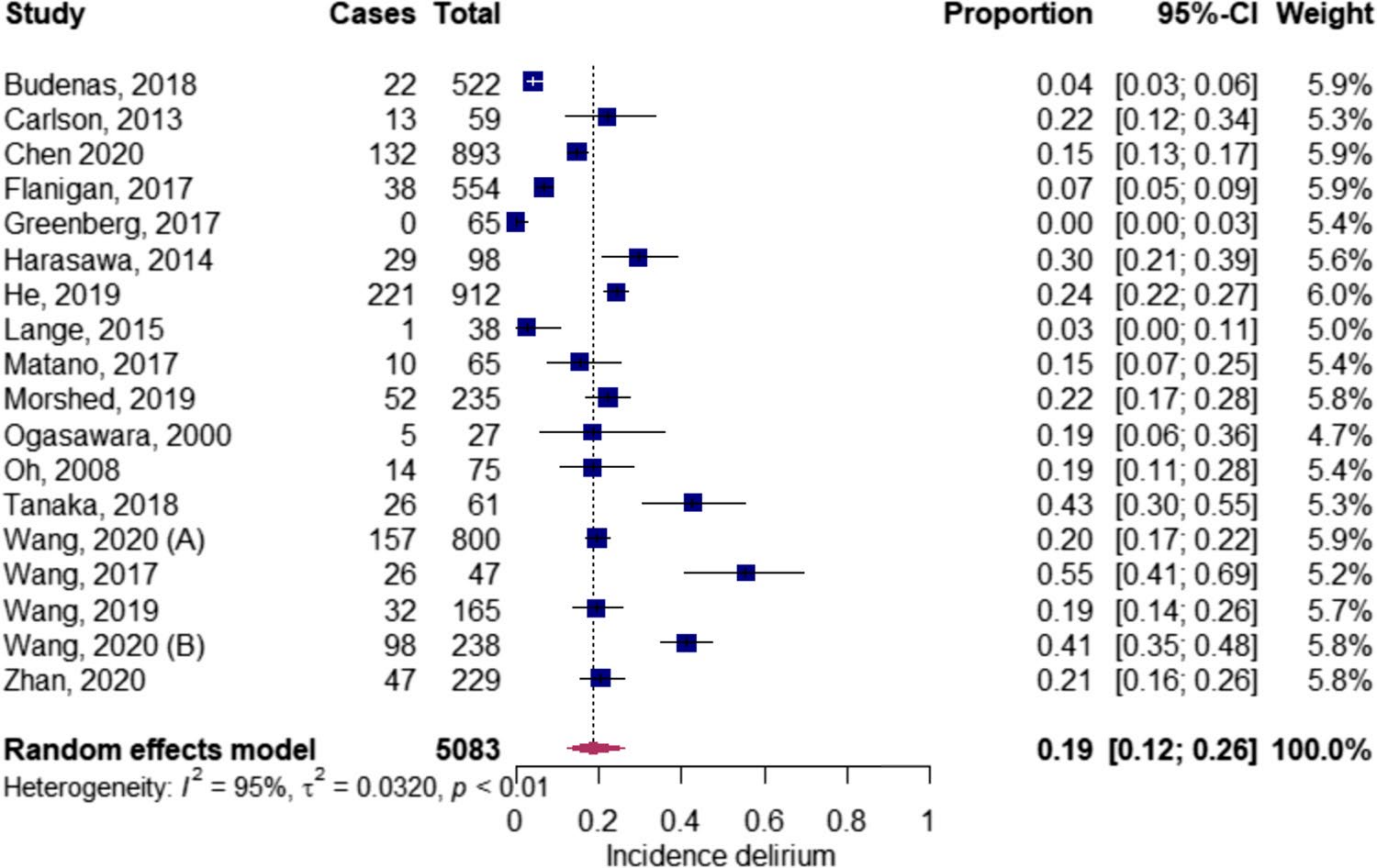
Pooled incidence delirium in neurosurgery

**Fig. 2:**
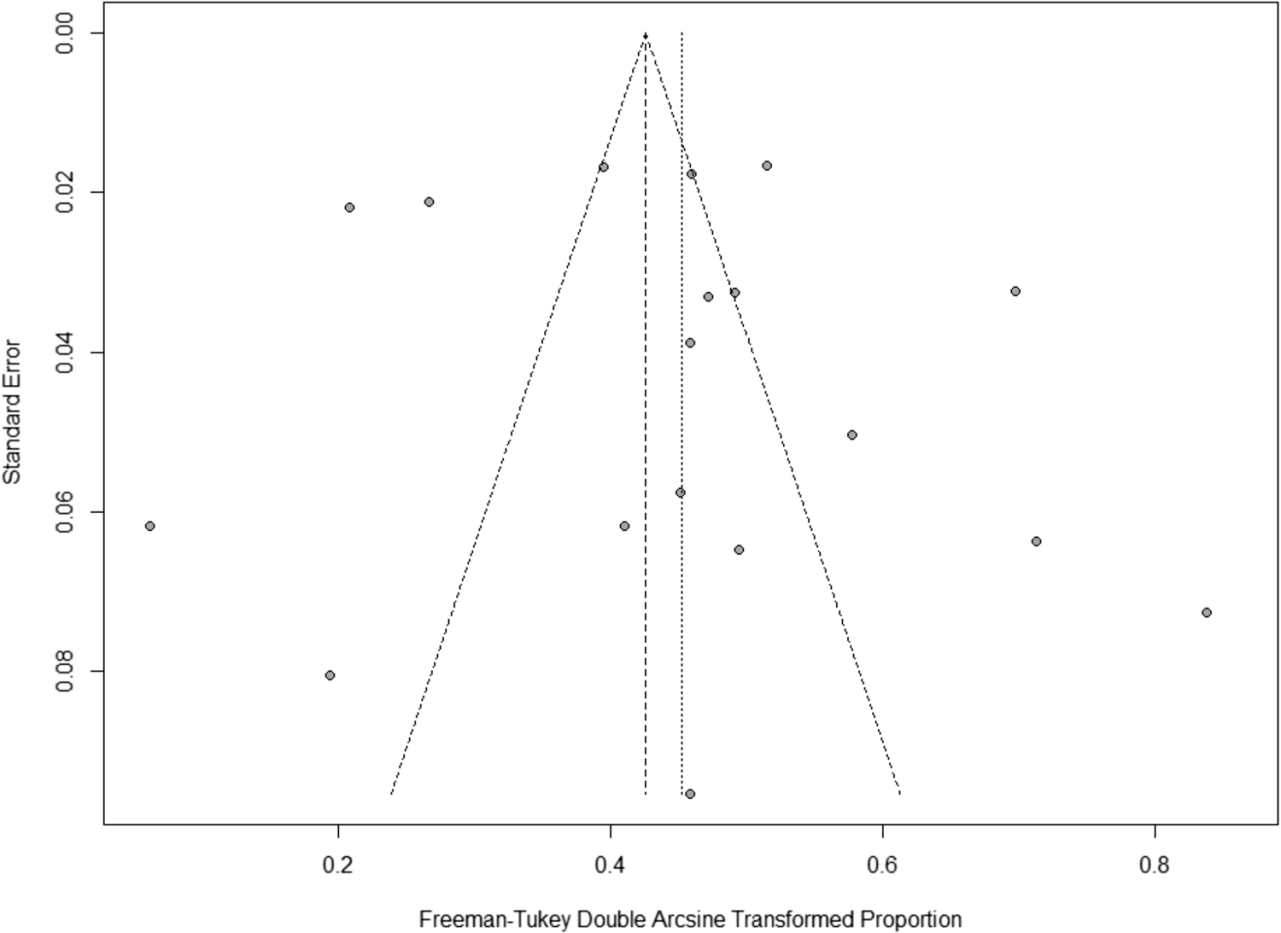
Funnel plot pooled incidence delirium in neurosurgery

One study did not report the incidence of delirium within the operated population [48]. Meta-analysis was conducted for 18 studies, after excluding one outlying study (Appendix 4), [54] resulting in a pooled incidence of post-operative delirium after intracranial surgery of 19.0% (*n*_p_ = 5083; 0.19; CI 0.12–0.26) (Figs. 1 and 2) [5, 7, 9–11, 32, 44–47, 49–53, 55, 56]. The mean/SD of onset in days, reported in three studies, was 2.8/0.6 [9, 45, 53]. Four studies, distinguishing the delirium subtypes, reported the hypoactive form in 38.9–68.1%, hyperactive form in 17.2–50.8% and the mixed form in 7.57–29.6% of the patients [4, 5, 53, 55].

### Subgroup analysis

#### Delirium assessment tools

**Fig. 3:**
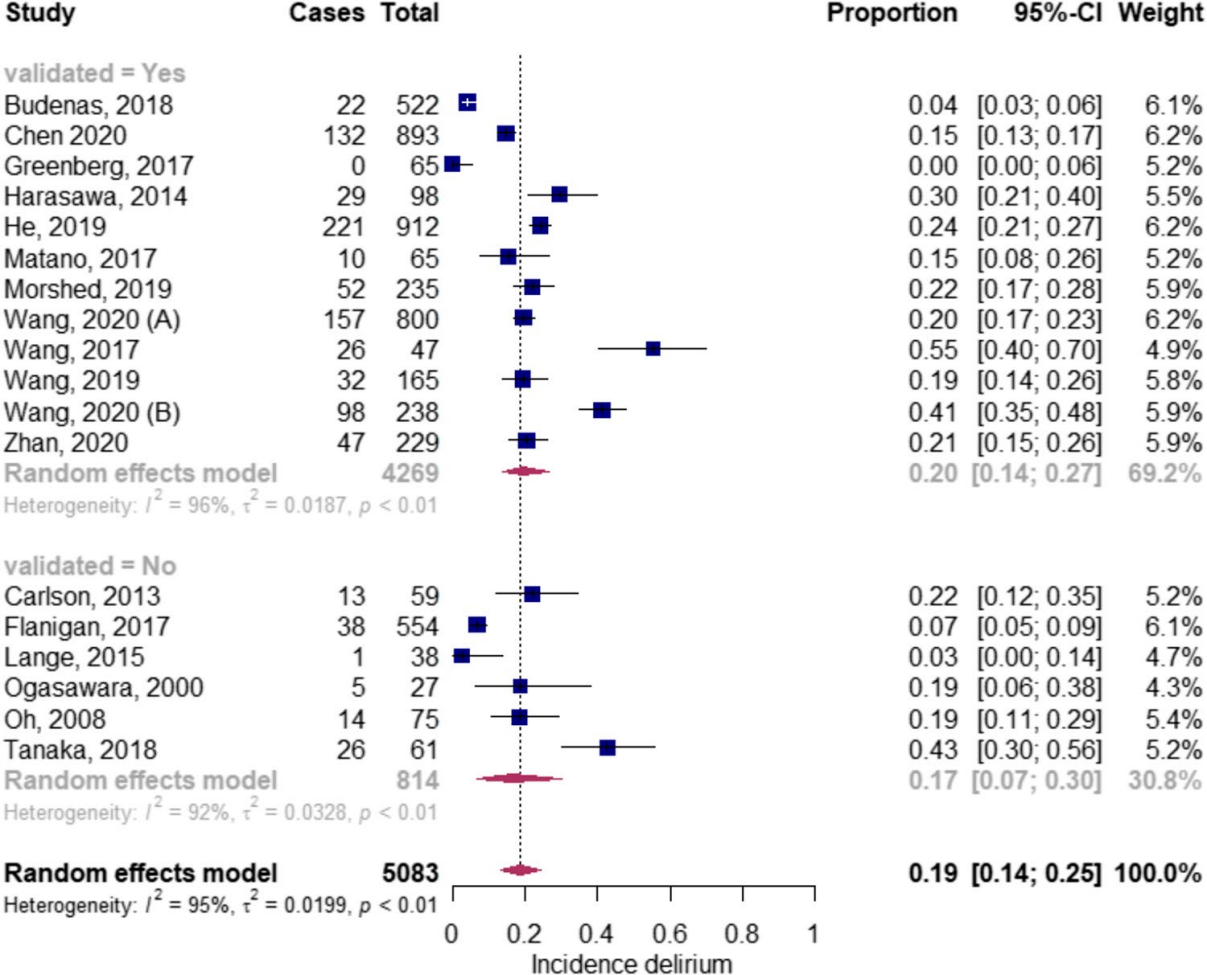
Subgroup analysis validated vs non-validated screening tools

The incidence of delirium in studies using validated tools and non-validated tools was 20.0% (*n*_p_ = 4269; 0.20; CI 0.14–0.27) [7, 9, 10, 32, 44, 46, 47, 51, 52, 55] and 17.0%, respectively (*n*_p_ = 814; 0.17; CI 0.07–0.30) (Fig. 3) [5, 11, 45, 49, 50, 56, 57]. The delirium incidence rates were 19.0%, 15.0%, 24.0% and 30.0% when using the CAM(-ICU), [4, 7, 32, 44, 46–48, 51, 53, 55] ICDSC, [10, 39] DOS [9, 43] and NEECHAM, [52] respectively.

#### Frequency and follow-up of daily delirium assessment

Pooled analysis of studies which did not report frequency of delirium assessment resulted in an incidence of 18.0% (*N* = 2746; 0.18; CI 0.11–0.25), [5, 7, 9, 11, 23, 44–46, 49, 50, 52, 56] 20.0% (*n*_p_ = 1029; 0.20; CI 0.17–0.22) [46, 47, 51, 57] in case of screening once per day, 36.0% (*N* = 350; 0.36; CI 0.17–0.57) [10, 46–48, 53] in case of screening twice per day and 5.0% (*n*_p_ = 958; 0.05; CI 0.00–0.28) [8, 32] in case of screening three times per day. Pooled analysis of studies assessing delirium during < 3 days resulted in an incidence of 18.0% (*n*_p_ = 3775; 0.18; CI 0.12–0.24) [5, 7, 9, 11, 32, 44–46, 49–52, 56, 57] and in 21.0% (*n*_p_ = 1308; 0.21; CI 0.07–0.40) in case of ≥ 3 days [4, 5, 7, 9–11, 32, 39, 43–45, 47–50, 52, 53, 55].

#### Clinical features

The pooled analysis of patients undergoing craniotomy (i.e. requiring bone flap removal) led to a delirium incidence of 15.0% (*n*_p_ = 2954; 0.15; CI 0.04–0.32) [7, 9, 23, 32, 52, 53, 55]. The incidence of delirium varied per type of neurosurgical disease; the incidence of 8.0% in neuro-oncologic patients (*n*_p_ = 1969; 0.08; CI 0.03–0.15), [7, 23, 55] 20% in functional neurosurgical patients (*n*_p_ = 552; 0.20; 0.12–0.30), 24.0% in microvascular decompression patients (*n*_p_ = 912; 0.24; CI 0.22–0.27), [9] 19.0% in TBI patients (*n*_p_ = 27;0.19; CI 0.06–0.36), [56, 57] 42.0% in neurovascular patients (*n*_p_ = 145; 0.42; CI 0.18–0.67) [52, 53] and 17.0% in the mixed neurosurgical population (*n*_p_ = 1478; 0.17; CI 0.09–0.28) [4, 10, 32, 39, 43–45, 47, 48]. Delirium incidence in patients admitted to the ICU, ward or both were respectively 24.0% (*n*_p_ = 1150; 0.24; CI 0.08–0.46), [4, 5, 7, 9–11, 32, 39, 43, 45, 47, 49, 50, 52, 53, 56, 57], 17.0% (*n*_p_ = 2805; 0.17; 0.11–0.25) [7, 9–11, 23, 45, 46, 49–52, 56] and 18.0% (*n*_p_ = 1128; 0.19;0.11–0.26) [4, 32, 44, 47, 48, 53, 55].

### Risk factors and health outcome

#### Risk factors

Independent risk factors from eight studies are presented in Table 3. Age was reported as significant risk factor in four, [7, 44, 46, 55] male gender in three [9, 51, 55] and sleep disturbances [9, 46] and longer surgery duration in two studies [4, 55]. All other risk factors were each described in only one study.

**Table 3:** Risk factors

Risk factors	Author	Odd’s ratio (OR)	95% CI	*p* value
**Reported in multiple studies**				
**Age**	Budenas, 2018	4.6	1.7–12.1	0.002
	Morshed, 2019	1.05	1.01–1.08	0.006
	Wang (A) 2020	1.0	1.02–1.06	< 0.001
	Chen 2020	1.80	1.01–1.04	< 0.001
**Sleep disturbance**	He, 2019	4.95	2.95–8.29	< 0.001
	Wang 2019	0.058	0.051–0.067	0.021
**Male gender**	Chen, 2020	1.80	1.01–1.04	< 0.001
	He, 2019	2.66	1.91–3.71	< 0.001
	Zhan, 2020	2.02	1.04–3.96	0.039
**Surgery duration**	Chen, 2020	2.51	1.67–3.76	< 0.001
	Wang(A), 2020	1.00—1.01	0.016	1.00
**Reported in single study**				
**Lesser than secondary education**	Budenas, 2018	3.5	1.3–9.1	0.011
**Poor functional status**		4.7	1.9–11.8	0.001
**Low haemoglobin**		5	1.1–22.5	0.036
**Off duty**	Chen, 2020	1.67	1.15–2.43	0.007
**Tobacco use history**		2.38	1.40–4.04	0.001
**Electrolyte disturbance**		1.67	1.15–2.43	0.007
**Temp > 38.5**		6.18	2.23–17.14	< 0.001
**Duration anaesthesia**		2.58	1.56–4.28	< 0.001
**Meningioma pathology**		0.57	0.34–0.96	0.036
**Pituitary adenoma**		0.32	0.17–0.59	< 0.001
**Subtentorial**		0.59	0.36–0.97	0.039
**Saddle area**		0.40	0.25–0.63	< 0.001
**Hypertension**	He, 2019	2.25	1.53–3.30	< 0.001
**Mount Fuji sign**		3.24	2.10–4.99	< 0.001
**Severe white matter lesions (Fazekas classifications 2 and 3)**	Matano, 2017	15	2–134	0.001
**Surrounding monitor**		6	1–32	0.001
**Surrounding delirium patients**		14	2–75	0.026
**Presence neurologic deficit**	Morshed, 2019	5.31	1.87–15.11	0.002
**Length of ICU stay**		1.23	1.07–1.43	0.004
**Non-motor symptoms scale of PD (NMSS)**	Wang, 2019	8.191	5.629–11.917	0.002
**Unified Parkinson’s disease rating scale (UPDRS III)**		2.284	1.614–3.232	0.047
**Preoperative length of stay**		1.230	1.053–1.437	0.009
**Preoperative brain atrophy**		3.912	3.597–4.255	0.038
**Non-motor symptoms scale of PD (NMSS)**		8.191	5.629–11.917	0.002
**Benign tumour**^**1**^	Wang, 2020A			
**Malignant tumour**^**1**^		2.82	1.52–4.88	< 0.001
**Frontal approach craniotomy**		3.01	1.79–5.05	< 0.001
**Duration surgery**				
**Episode of SpO2 < 90% at ICU admission**		8.22	1.38–48.92	0.021
**Emergence delirium, inadequate**^**2**^		11.15	4.8–25.88	< 0.001
**Emergence delirium, hyperactive**^**2**^		14.60	5.4–39.45	< 0.001
**Emergence delirium, hypoactive**^**2**^		11.64	7.75–20.10	< 0.001
**NRS for pain**		1.19	1.02–1.38	0.028
**Immobilizing factor**		1.64	1.3–2.08	< 0.001
**Cerebrovascular disease**		3.2	1.57–6.53	0.001
**Parkinson’s disease sleep scale PDSS)**	Zhan, 2020	0.984	0.97–0.99	0.034
**Preoperative cerebral ischaemia**		2.127	1.05–5.06	0.035
**preoperative pulmonary inflammation**		2.295	1.04–5.08	0.04
**Preoperative length of stay**		1.162	1.002–1.349	0.048

^1^Compared to benign tumour. ^2^Compared to non-emergence delirium

#### Meta-regression

**Fig. 4:**
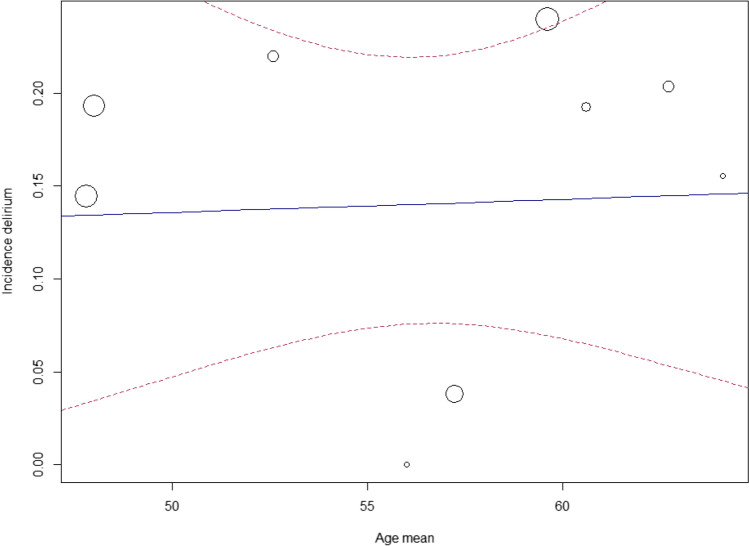
Meta-regression: age and incidence delirium

**Fig. 5:**
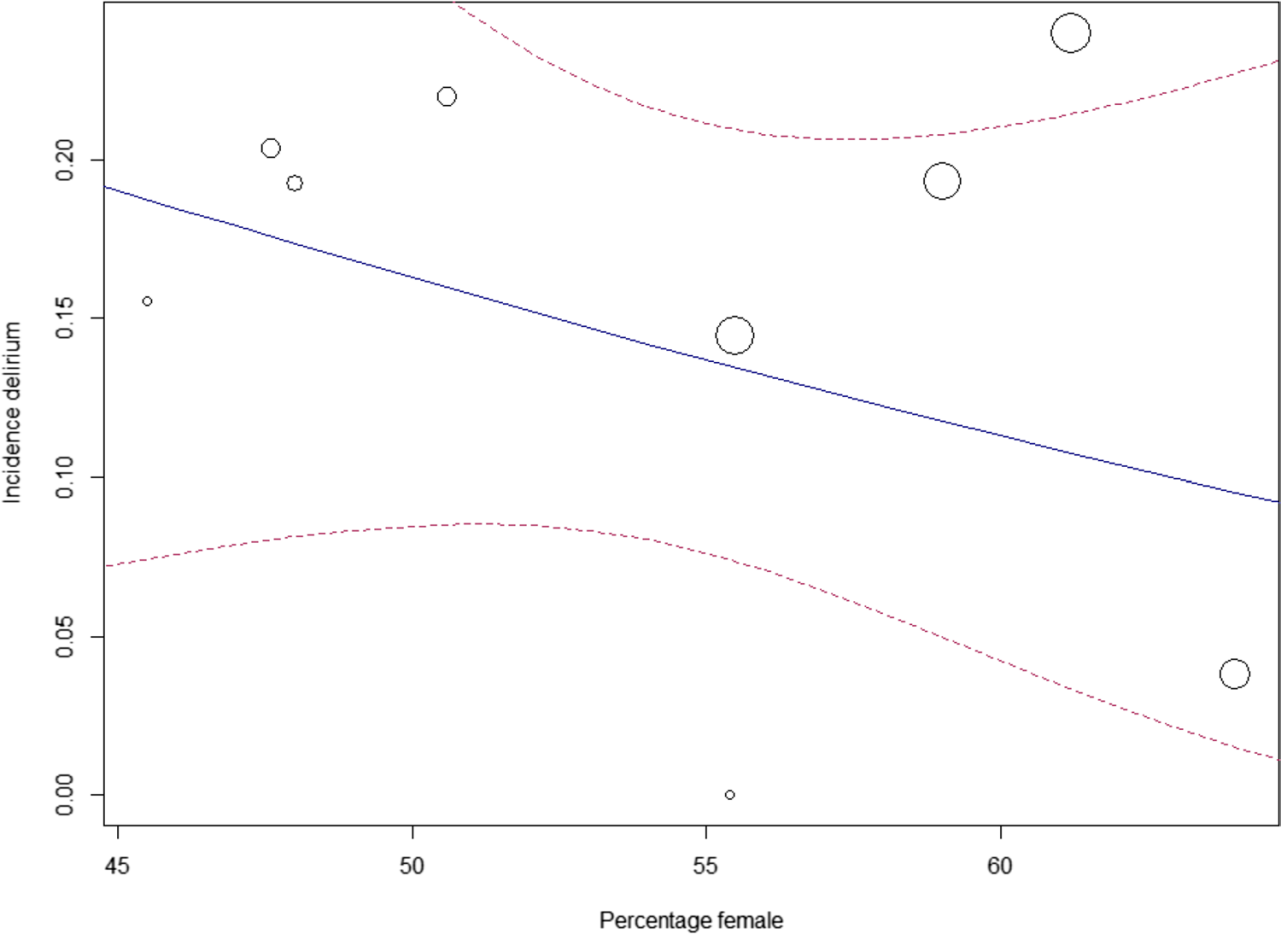
Meta-regression: gender and incidence delirium

Meta-regression was performed for age and gender (from baseline characteristics), for which no significant correlation was found with delirium occurrence (*p* = 0.91, respectively *p* = 0.37) (Figs. 4 and 5) [4, 7, 9, 10, 32, 43, 44, 46–48, 51–53, 55].

#### Health outcomes

Health outcomes were assessed in four studies. Table 4 illustrates health outcomes related to delirium. Delirium was significantly associated with restraint/fixation of patients in three studies [10, 53, 55, 58] and with an unfavourable Glasgow Outcome Scale at discharge [7], increased length of ICU, catheterization and disease in one study [55].

**Table 4:** Health outcomes

Health outcome	Author	Odd’s ratio (OR)	95% CI	*p* value
**Reported in multiple studies**				
**Patient restraint/fixation**	Chen, 2020	4.73	3.17–7.05	< 0.001
	Matano, 2017	8	1–75	0.001
	Wang 2017	22.51	5.25–96.49	0.000
**Reported in single study**				
**Unfavourable functional outcome**	Budenas, 2018	5.3	2.1–13.4	0.0005
**Length ICU**	Chen, 2020		1.10	1.00–1.20
**Time urinary catheterization**			1.05	1.01–1.08
**Length disease**			0.98	0.97–1.00

### Risk of bias

An overview of the risk of bias assessment is presented in Appendix 5. The quality of evidence was considered poor to moderate. The risk of bias in the study of Greenberg et al. [32] was considered with ‘some concerns’ due to unclear allocation concealment and missing data. The risk of bias in the study of Mokhtari et al. [48] was considered high due to incomplete data and exclusion of patients admitted to the ICU after randomization.

The quality of evidence in the cohort studies was poor in 11 (55.0%) studies, fair in three (15.0%) studies and good in six (30.0%) studies. Only three studies assessed delirium at baseline [21, 52, 55, 56]. Inter-observer reliability between the two researchers (PK/EK) for the NOS was ‘moderate’ (Cohen’s kappa (range); 0.62 (0.50–0.73)) 16].

### GRADE certainty rating

*The quality of evidence* was moderate for the studies included in the meta-analysis. *Imprecision* was considered moderate since the 95%CI was wide. *Inconsistency* was considered high since the 95%CI of the individual studies in the meta-analysis did not all overlap, which is confirmed by the heterogeneity test (*I*^2^ = 95.0%, *p* < 0.01). The risk for *indirectness* was considered moderate; although the type of neurosurgical patients included (neuro-oncology, neurovascular etc.) did differ, delirium was investigated in the population of interest. The risk for publication bias is considered high illustrated by the asymmetrical scattering in the funnel plot (Fig. 2). Based on the previous, the GRADE certainty rating is low to moderate.

## Discussion

To our knowledge this is the first systematic review and meta-analysis studying delirium in patients undergoing intracranial surgery. We found an overall incidence of 19%, but the diagnostic method to assess the presence of delirium and the type of neurosurgical patients were highly variable. Although the incidence rate is significant, the current evidence is too limited to draw firm conclusions on risk factors and health outcomes associated with delirium in this specific group of patients.

In this review, it was not possible to investigate which delirium assessment tool was most suitable for the neurosurgical population, since diagnostic accuracy was not determined in any of the included studies and no specific reference standard exists for this population, apart from the DSM criteria. The CAM was mostly used as a screening tool, which is considered a reliable assessment instrument for delirium in postsurgical patients [59]. The second most used assessment tool in this review was the ICDSC, a tool primarily developed for the ICU [60]. The CAM-ICU has a higher sensitivity and specificity compared to the ICDSC (80% and 96%, respectively, 74% and 82%) in critically ill patients [61], which might explain the slightly higher incidence (CAM-ICU; 19%, ICDSC; 15%). Future studies should further validate these screening tools as certain symptoms specific to the neurosurgical patient overlap with diagnostic criteria of delirium.

A considerable proportion of the studies in our review used non-validated tools [5, 11, 21, 45, 49, 50, 56, 57]. Most of these studies were retrospective with delirium assessment based on ‘positive’ symptoms [11]. These assessments might fail to recognizing delirium, especially the hypoactive type which compromises 26–58% of delirium in this population [4, 5, 23, 53]. Structured screening done once vs twice per day increased the incidence (20.0 vs 36.0%), but in studies screening three times per day, the incidence surprisingly decreased (5.0%). This might have been caused by one study, reporting 0% incidence with short follow-up time (within 24 h) [32]. Still, future studies should assess delirium at several moments per day, as delirium fluctuates and infrequent assessments might falsely decrease delirium detection [1].

In our study, post-operative delirium after intracranial surgery occurred in 19% (range 5%–37%), comparable to the pooled incidence (12–43%) reported by Patel et al., evaluating delirium in neurocritical care patients [62]. The difference in incidence between the ICU compared to the ward was not as large as we expected (24.0 vs 17.0%). Explanations for this might include all ICU patients were diagnosed with a valid delirium assessment tool, as opposed to only half of the patients on the ward, and use of sedatives might artificially decrease the incidence of delirium since in drug-induced coma, delirium is by definition undetectable. The clear criteria of validated delirium screening tools compared to the more loose non-validated criteria in many other studies might have affected these incidence rates.

The highest incidence of delirium was found in patients undergoing neurovascular surgery (42%) [52, 53]. A possible explanation for this may be cerebral ischaemia, hypoxia and oxidative stress, induced by, e.g. temporary clipping and bypass techniques, which are described as mechanisms in the pathophysiology of delirium [2]. Moreover, neurovascular procedures are often characterized by a relative long duration of anaesthesia and require frequent post-operative sedation and mechanical ventilation [4, 43]. A relatively lower incidence was observed in the TBI study, possibly caused by the low surgical invasiveness in this cohort, as only patients undergoing burr hole drainage without craniotomy (i.e. requiring boneflap removal) were included [56].

We did not find a correlation between age and delirium, in contrary to literature in other populations [63]. An explanation might be the relatively low range in age (47.8–64.1 years) of the patients in the studies, which is representative of the neurosurgical population. Moreover, the meta-regression analysis might have been underpowered due to high heterogeneity [64]. On the other hand, age might be a less relevant factor after intracranial surgery as it was only described as a risk factor for delirium in four studies [7, 44, 46, 55] and not confirmed in the other five studies [9, 10, 38, 46].

### Limitations

The most important limitation in our study is the high heterogeneity of our included studies caused by the differences in delirium assessment methods and clinical differences. Moreover, scattering in the funnel plot indicates a high probability of publication bias. Hence, the findings, especially the quantitative analysis, of this review should be interpreted carefully and be regarded as hypothesis-generating.

### Future research

Future research should assess delirium at several moments per day and focus on the validation of structural delirium assessment tools and the prognostic relevance of delirium for clinical outcomes and surgical complications in neurosurgical patients. This is desirable before interventional trials are undertaken to assess optimal management. Furthermore, our analyses indicate that the definition of delirium after intracranial surgery requires consensus to enhance further research. Further, details on depth and length of anaesthesia for surgical procedures and timing of delirium assessments relative to the surgery should be taken into account, to distinguish anaesthesia effects from the impact of structural cerebral pathologies on the phenomenology of delirium.

## Conclusion

This is the first systematic review and meta-analysis on delirium after intracranial surgery in neurosurgical patients. Delirium is a frequently occurring adverse event in the neurosurgical clinical practice, but limited consensus exists on the diagnostic criteria. Future research should focus on validating delirium assessment methods in the neurosurgical population and define the prognostic impact of delirium.

## Supplementary Information

Below is the link to the electronic supplementary material.

Supplementary file1 (DOCX 2203 KB)

Supplementary file2 (PDF 248 KB)

## Data Availability

Data is available and may be requested.
