# Supplementary material for: Delirium in neurosurgery: a systematic review and meta-analysis
Source: Neurosurg Rev. 2021 Aug 16;45(1):329–41. doi: 10.1007/s10143-021-01619-w (PMC8827408; doi:10.1007/s10143-021-01619-w)

**Appendix A.**

Delirium neurosurgery Date of search: 31^st^ March, 2021

| Embase.com | 3104 | 3076 |
| --- | --- | --- |
| Medline Ovid | 1699 | 426 |
| Web of science Core Collection | 1366 | 383 |
| PsycINFO Ovid | 293 | 104 |
| Cochrane central register of trials | 512 | 301 |
| **Total** | **6974** | **4290** |

**Embase.com**

('delirium'/de OR 'postoperative delirium'/de OR 'postoperative cognitive dysfunction'/de OR 'intensive care psychosis'/de OR 'drug induced psychosis'/de OR (('postoperative complication'/de OR 'postoperative period'/de) AND ('mental disease'/de OR 'confusion'/exp OR 'agitation'/exp OR 'agitation assessment'/exp OR 'emergence agitation'/de OR 'cognitive defect'/exp OR cognition/de OR 'neuropsychiatry'/exp OR 'psychosis'/de OR Delusion/de OR hallucination/de)) OR (delirium* OR confusion* OR agitat* OR ((cognit* OR mental-change* OR neuropsychiatr* OR Encephalopath* OR Mental-alteration* OR Hypoactiv* OR Hyperactiv*) NEAR/3 (postop* OR post-op* OR complication* OR intensive-care OR icu)) OR ((cognit* OR mental-change* OR neuropsychiatr*) NEAR/3 (after OR following) NEAR/3 (surg* OR resect*))):ab,ti) AND ('neurosurgery'/de OR 'auditory brain stem implantation'/exp OR 'nerve surgery'/exp OR 'neuroendoscopy'/exp OR 'neuronavigation'/exp OR 'skull surgery'/exp OR 'ventriculostomy'/exp OR 'brain cancer'/exp/dm_su OR 'glioma'/exp/dm_su OR 'brain depth stimulation'/de OR (neurosurg* OR Trephin* OR craniectomy OR cranioplasty OR craniotom* OR ((brain* OR cerebr* OR cerebell* OR nerve* OR cranial* OR skull* OR hydrocephal* OR glioma OR glioblastoma* OR meningi* OR gangliogliom* OR schwannom* OR astrocytom* OR Acoustic-neurom* OR Chordom* OR Lymphom* OR Craniopharyngiom* OR Ependymom* OR Medulloblastom* OR epilep* OR posterior-fossa* OR subdural* OR epidural* OR subarachnoidal* OR intraparenchym* OR intra-parenchym* OR ventricul* OR neurooncol* OR neuro-oncol* OR frontal-lobe* OR parietal-lobe* OR occipital-lobe* OR temporal-lobe* OR Intracranial) NEAR/6 (surg* OR operat* OR postsurg* OR postoperat* OR drain* OR shunt* OR decompressi*)) OR ventriculostom* OR lobectom* OR (brain NEAR/3 (depth OR deep) NEAR/3 stimulat*) OR craniosynostos*):ab,ti) NOT ([Conference Abstract]/lim OR [letter]/lim OR [editorial]/lim) NOT ('case report'/de OR 'case report':ti) NOT ([animals]/lim NOT [humans]/lim) AND [English]/lim

**Medline Ovid**

(Delirium/ OR Psychoses, Substance-Induced/ OR ((Postoperative Complications/ OR Postoperative Period/) AND (Mental Disorders/ OR Confusion/ OR Emergence Delirium/ OR Cognition/ OR Neuropsychiatry/ OR Psychotic Disorders/ OR Delusions/ OR Hallucinations/)) OR (delirium* OR confusion* OR agitat* OR ((cognit* OR mental-change* OR neuropsychiatr* OR Encephalopath* OR Mental-alteration* OR Hypoactiv* OR Hyperactiv*) ADJ3 (postop* OR post-op* OR complication* OR intensive-care OR icu)) OR ((cognit* OR mental-change* OR neuropsychiatr*) ADJ3 (after OR following) ADJ3 (surg* OR resect*))).ab,ti.) AND (Neurosurgery/ OR exp Neurosurgical Procedures/ OR Auditory Brain Stem Implantation/ OR Neuroendoscopy/ OR Neuronavigation/ OR Ventriculostomy/ OR exp Brain Neoplasms/su OR Glioma/su OR Craniotomy/ OR (neurosurg* OR Trephin* OR craniectomy OR cranioplasty OR craniotom* OR ((brain* OR cerebr* OR cerebell* OR nerve* OR cranial* OR skull* OR hydrocephal* OR glioma OR glioblastoma* OR meningi* OR gangliogliom* OR schwannom* OR astrocytom* OR Acoustic-neurom* OR Chordom* OR Lymphom* OR Craniopharyngiom* OR Ependymom* OR Medulloblastom* OR epilep* OR posterior-fossa* OR subdural* OR epidural* OR subarachnoidal* OR intraparenchym* OR intra-parenchym* OR ventricul* OR neurooncol* OR neuro-oncol* OR frontal-lobe* OR parietal-lobe* OR occipital-lobe* OR temporal-lobe* OR Intracranial) ADJ6 (surg* OR operat* OR postsurg* OR postoperat* OR drain* OR shunt* OR decompressi*)) OR ventriculostom* OR lobectom* OR (brain ADJ3 (depth OR deep) ADJ3 stimulat*) OR craniosynostos*).ab,ti.) NOT (news OR congres* OR abstract* OR book* OR chapter* OR dissertation abstract*).pt. NOT (case reports/ OR case report.ti.) NOT (exp animals/ NOT humans/) AND english.la.

**PsycINFO Ovid**

(Delirium/ OR ((Postsurgical Complications / OR Surgical Patients /) AND (Mental Disorders/ OR Mental Confusion / OR Cognition/ OR Neuropsychiatry/ OR Psychosis/ OR Delusions/ OR Hallucinations/)) OR (delirium* OR confusion* OR agitat* OR ((cognit* OR mental-change* OR neuropsychiatr* OR Encephalopath* OR Mental-alteration* OR Hypoactiv* OR Hyperactiv*) ADJ3 (postop* OR post-op* OR complication* OR intensive-care OR icu)) OR ((cognit* OR mental-change* OR neuropsychiatr*) ADJ3 (after OR following) ADJ3 (surg* OR resect*))).ab,ti.) AND (Neurosurgery/ OR (neurosurg* OR Trephin* OR craniectomy OR cranioplasty OR craniotom* OR ((brain* OR cerebr* OR cerebell* OR nerve* OR cranial* OR skull* OR hydrocephal* OR glioma OR glioblastoma* OR meningi* OR gangliogliom* OR schwannom* OR astrocytom* OR Acoustic-neurom* OR Chordom* OR Lymphom* OR Craniopharyngiom* OR Ependymom* OR Medulloblastom* OR epilep* OR posterior-fossa* OR subdural* OR epidural* OR subarachnoidal* OR intraparenchym* OR intra-parenchym* OR ventricul* OR neurooncol* OR neuro-oncol* OR frontal-lobe* OR parietal-lobe* OR occipital-lobe* OR temporal-lobe* OR Intracranial) ADJ6 (surg* OR operat* OR postsurg* OR postoperat* OR drain* OR shunt* OR decompressi*)) OR ventriculostom* OR lobectom* OR (brain ADJ3 (depth OR deep) ADJ3 stimulat*) OR craniosynostos*).ab,ti.) NOT (news OR congres* OR abstract* OR book* OR chapter* OR dissertation abstract*).pt. NOT (case reports/ OR case report.ti.) NOT (exp animals/ NOT humans/) AND english.la.

**Web of science Core Collection**

TS=(((delirium* OR confusion* OR agitat* OR ((cognit* OR mental-change* OR neuropsychiatr* OR Encephalopath* OR Mental-alteration* OR Hypoactiv* OR Hyperactiv*) NEAR/2 (postop* OR post-op* OR complication* OR intensive-care OR icu)) OR ((cognit* OR mental-change* OR neuropsychiatr*) NEAR/2 (after OR following) NEAR/2 (surg* OR resect*)))) AND ((neurosurg* OR Trephin* OR craniectomy OR cranioplasty OR craniotom* OR ((brain* OR cerebr* OR cerebell* OR nerve* OR cranial* OR skull* OR hydrocephal* OR glioma OR glioblastoma* OR meningi* OR gangliogliom* OR schwannom* OR astrocytom* OR Acoustic-neurom* OR Chordom* OR Lymphom* OR Craniopharyngiom* OR Ependymom* OR Medulloblastom* OR epilep* OR posterior-fossa* OR subdural* OR epidural* OR subarachnoidal* OR intraparenchym* OR intra-parenchym* OR ventricul* OR neurooncol* OR neuro-oncol* OR frontal-lobe* OR parietal-lobe* OR occipital-lobe* OR temporal-lobe* OR Intracranial) NEAR/5 (surg* OR operat* OR postsurg* OR postoperat* OR drain* OR shunt* OR decompressi*)) OR ventriculostom* OR lobectom* OR (brain NEAR/2 (depth OR deep) NEAR/2 stimulat*) OR craniosynostos*))) AND DT=(article) AND LA=(english)

**Cochrane central register of trials**

((delirium* OR confusion* OR agitat* OR ((cognit* OR mental-change* OR neuropsychiatr* OR Encephalopath* OR Mental-alteration* OR Hypoactiv* OR Hyperactiv*) NEAR/3 (postop* OR post-op* OR complication* OR intensive-care OR icu)) OR ((cognit* OR mental-change* OR neuropsychiatr*) NEAR/3 (after OR following) NEAR/3 (surg* OR resect*))):ab,ti) AND ((neurosurg* OR Trephin* OR craniectomy OR cranioplasty OR craniotom* OR ((brain* OR cerebr* OR cerebell* OR nerve* OR cranial* OR skull* OR hydrocephal* OR glioma OR glioblastoma* OR meningi* OR gangliogliom* OR schwannom* OR astrocytom* OR Acoustic-neurom* OR Chordom* OR Lymphom* OR Craniopharyngiom* OR Ependymom* OR Medulloblastom* OR epilep* OR posterior-fossa* OR subdural* OR epidural* OR subarachnoidal* OR intraparenchym* OR intra-parenchym* OR ventricul* OR neurooncol* OR neuro-oncol* OR frontal-lobe* OR parietal-lobe* OR occipital-lobe* OR temporal-lobe* OR Intracranial) NEAR/6 (surg* OR operat* OR postsurg* OR postoperat* OR drain* OR shunt* OR decompressi*)) OR ventriculostom* OR lobectom* OR (brain NEAR/3 (depth OR deep) NEAR/3 stimulat*) OR craniosynostos*):ab,ti)

**Appendix B: the Newcastle-Ottawa Scale criteria.**

| **Criteria^1^** | **Acceptable^2^ (star awarded):** | **Unacceptable^2^ (star not awarded):** |
| --- | --- | --- |
| *Selection: representativeness of exposed cohort* | Entire study must represent neurosurgical cohort or adequately specify in case of mixed group. | Neurosurgical cohort mixed with other types of patients, not further specified. |
| *Selection: representativeness of non-exposed cohort* | Same setting as exposed (delirium) cohort. | Different setting from exposed (delirium) cohort. |
| *Selection:*  *ascertainment of exposure* | Ascertainment of delirium must be through a completely clear scoring system (i.e. three out of four CAM features). | Delirium assessment definition without clear scoring system. |
| *Selection:*  *demonstration outcome interest not present at start study* | Must be stated that delirium was excluded at baseline/before operation. | No exclusion of delirium at baseline (or before operation) or no statement on this. |
| *Comparability* | Type of neurosurgical intervention must be described and comparable between delirium and non-delirium group. | Type of neurosurgical intervention undefined or incomparable between delirium and non-delirium group. |
|  | Timing of delirium assessment must be similar between delirium and non-delirium group. | Different timing of delirium assessment between groups or no clear definition/statement on this. |
| *Exposure: follow up duration* | Delirium assessment procedure: validated delirium screening + DSM criteria by two independent and blinded researchers. | Delirium definition procedure otherwise. |
| *Exposure: adequacy follow-up* | Follow-up of delirium from date of craniotomy up to least 3 days. | Shorter follow-up delirium. |
| *Exposure: non-response rate* | In case of missing data: amount of missed data must be similar between the delirium and non-delirium group. | Significant difference in missing data between the delirium and non-delirium group or more than 10% of the entire sample size. |
| 1. Criteria of the Newcastle Ottawa Scale. 2. Adaption of the criteria for quality appraisal of the included studies in this review. | | |

**Appendix C. PRISMA Flowchart**

## Identification

## Eligibility

## Included

## Screening

Full-text articles excluded, with reasons
(n_s_ = 27)

- Not reproducible delirium definition (n_s_ = 9)

- Abstract only (n_s_ = 3)

- Additional duplicate (n_s_ = 3)

- Pediatric patient in cohort (n_s_ = 1)

- Overlapping population with an included study (n_s_ = 3)

- No assessment of delirium (n_s_ =1)

- No original data (n_s_ = 2)

- Unclear number of intracranial surgery (n_s_ = 5)

Records screened on title/abstract
(n_s_ = 4290)

Records excluded
(n_s_ = 4243)

Studies included in quantitative synthesis (meta-analysis)

(n_s_ = 18)

Records after duplicates removed
(n_s_ = 4290)

Studies included in qualitative synthesis
(n_s_ = 20)

Full-text articles assessed for eligibility
(n_s_ = 47)

Records identified through database searching
(n_s_ = 6974)

**Appendix D: overview of risk of bias assessment**

**Risk of Bias: Cochrane Risk of Bias tool for Randomized Controlled Trials**

| Study ID | Method of randomization | Allocation Concealment | Blinding participants | Blinding Assessors | Incomplete Outcome | Selection of reporting | Other Bias Adequately Assessed | Overall |
| --- | --- | --- | --- | --- | --- | --- | --- | --- |
| Greenberg, 2017 | Low | Unclear | Low | Low | Unclear | Low | Low | Some concerns |
| Mohktari, 2020 | Low | Unclear | Low | Low | High | Low | High | High |

**Risk of Bias: Newcastle-Ottawa Scale (NOS) for cohort-studies**

|  | Selection | | | | Comparability | | Exposure | | |  | |
| --- | --- | --- | --- | --- | --- | --- | --- | --- | --- | --- | --- |
|  | Representativeness cohort | Selection non-exposed | Ascertainment exposure | Outcome baseline | Specification groups | Frequent assessment | Ascertainment exposure | Adequacy follow-up | Non response rate | | Overall |
| Budenas, 2018 | ⋆ | ⋆ | ⋆ | - | N/A | - | - | - | - | | Poor |
| Carlson, 2014 | ⋆ | ⋆ | - | - | N/A | - | - | ⋆ | N/A | | Poor |
| Chen, 2020 | ⋆ | ⋆ | - | ⋆ | ⋆ | ⋆ | - | ⋆ | N/A | | Good |
| Flanigan, 2017 | ⋆ | ⋆ | - | - | N/A | - | - | ⋆ | N/A | | Poor |
| Harasawa, 2014 | ⋆ | ⋆ | ⋆ | ⋆ | N/A | ⋆ | - | ⋆ | ⋆ | | Good |
| He, 2019 | ⋆ | ⋆ | ⋆ | - | N/A | ⋆ | - | ⋆ | N/A | | Poor |
| Hosoya, 2018 | - | ⋆ | ⋆ | - | ⋆ | - | - | - | N/A | | Poor |
| Lange, 2015 | ⋆ | ⋆ | - | - | N/A | - | - | ⋆ | N/A | | Poor |
| Matano, 2017 | - | ⋆ | ⋆ | - | - | ⋆ | - | ⋆ | ⋆ | | Fair |
| Morshed, 2019 | ⋆ | ⋆ | ⋆ | - | ⋆ | - | - | ⋆ | - | | Poor |
| Ogasawara, 2020 | ⋆ | ⋆ | - | ⋆ | N/A | - | - | - | ⋆ | | Poor |
| Oh, 2008 | ⋆ | ⋆ | ⋆ | - | - | - | - | ⋆ | N/A | | Poor |
| Tanaka, 2018 | ⋆ | ⋆ | - | - | N/A | - | - | ⋆ | N/A | | Poor |
| Wang, 2020 (A) | ⋆ | ⋆ | ⋆ | - | ⋆ | ⋆ | - | ⋆ | ⋆ | | Good |
| Wang, 2017 | ⋆ | ⋆ | ⋆ | - | N/A | ⋆ | - | ⋆ | ⋆ | | Good |
| Wang, 2019 | ⋆ | ⋆ | ⋆ | - | N/A | - | - | - | N/A | | Poor |
| Wang, 2020 (B) | ⋆ | ⋆ | - | - | - | ⋆ | - | ⋆ | ⋆ | | Fair |
| Zhan, 2020 | ⋆ | ⋆ | ⋆ | - | ⋆ | ⋆ | - | - | ⋆ | | Good |
|  | | | | | | | | | |  | |

**Appendix E. identifying outlying and influential studies.**

1. Budenas, 2018

2. Carlson, 2013

3. Chen, 2020

4. Flanigan, 2017

5. Greenberg, 2017

6. Harasawa, 2014

7. He, 2019

8. Hosoya, 2018

9. Lange, 2015

10. Matano, 2017

11. Morshed, 2019

12. Ogasawara, 2000

13. Oh, 2008

14. Tanaka, 2018

15. Wang, 2020 (A)

16. Wang, 2017

17. Wang, 2019

18. Wang, 2020 (B)

19. Zhan, 2020

Z value above 3 indicates a statistical outlier (in red):

resid se z

8 0.6979 0.2039 3.4229

5 -0.4477 0.2257 -1.9831

16 0.3696 0.2373 1.5574

1 -0.2940 0.2326 -1.2639

9 -0.3072 0.2459 -1.2495

4 -0.2331 0.2373 -0.9826

14 0.2380 0.2445 0.9734

18 0.2223 0.2389 0.9308

3 -0.0971 0.2433 -0.3993

6 0.0955 0.2475 0.3858

10 -0.0810 0.2504 -0.3235

13 -0.0370 0.2501 -0.1478

17 -0.0303 0.2468 -0.1228

7 0.0296 0.2444 0.1213

15 -0.0292 0.2445 -0.1194

12 -0.0296 0.2608 -0.1136

19 -0.0161 0.2460 -0.0656

2 0.0075 0.2519 0.0299

11 0.0044 0.2460 0.0178

Leaving study 8 (Hosoya, 2018) out reveals the largest change in incidence (illustrated in red and with a red dot in the graph):

| estimate zval pval ci.lb ci.ub Q Qp tau2 I2 H2 |
| --- |
| 1 0.2291 9.2120 0.0000 0.1445 0.3260 326.9867 0.0000 0.0506 97.9407 48.5597 |
| 2 0.2153 8.5153 0.0000 0.1295 0.3153 440.3039 0.0000 0.0560 98.3251 59.7059 |
| 3 0.2201 8.6236 0.0000 0.1336 0.3206 435.8726 0.0000 0.0556 97.9727 49.3278 |
| 4 0.2263 8.9777 0.0000 0.1407 0.3249 374.3800 0.0000 0.0527 98.0072 50.1799 |
| 5 0.2346 9.9355 0.0000 0.1540 0.3260 405.1772 0.0000 0.0445 97.8997 47.6123 |
| 6 0.2114 8.4617 0.0000 0.1266 0.3106 432.6104 0.0000 0.0555 98.2947 58.6418 |
| 7 0.2143 8.4649 0.0000 0.1284 0.3145 409.5760 0.0000 0.0562 97.9837 49.5953 |
| 8 0.1877 10.3372 0.0000 0.1243 0.2603 373.4364 0.0000 0.0320 97.1216 34.7421 |
| 9 0.2282 9.1912 0.0000 0.1438 0.3251 432.6441 0.0000 0.0510 98.1736 54.7519 |
| 10 0.2191 8.6173 0.0000 0.1329 0.3194 441.1688 0.0000 0.0557 98.3132 59.2828 |
| 11 0.2154 8.4915 0.0000 0.1294 0.3158 437.6484 0.0000 0.0562 98.2588 57.4324 |
| 12 0.2168 8.5849 0.0000 0.1311 0.3166 441.1904 0.0000 0.0557 98.3297 59.8679 |
| 13 0.2173 8.5530 0.0000 0.1311 0.3176 441.1376 0.0000 0.0560 98.3184 59.4664 |
| 14 0.2053 8.5509 0.0000 0.1235 0.3010 421.4747 0.0000 0.0526 98.2198 56.1743 |
| 15 0.2170 8.5200 0.0000 0.1306 0.3176 438.1105 0.0000 0.0562 98.0273 50.6917 |
| 16 0.1999 8.7943 0.0000 0.1221 0.2908 409.5013 0.0000 0.0482 98.0671 51.7357 |
| 17 0.2170 8.5311 0.0000 0.1307 0.3175 440.7493 0.0000 0.0561 98.2855 58.3258 |
| 18 0.2055 8.5201 0.0000 0.1233 0.3016 370.1912 0.0000 0.0529 98.1522 54.1179 |
| 19 0.2164 8.5117 0.0000 0.1302 0.3169 439.6458 0.0000 0.0562 98.2610 57.5052 |


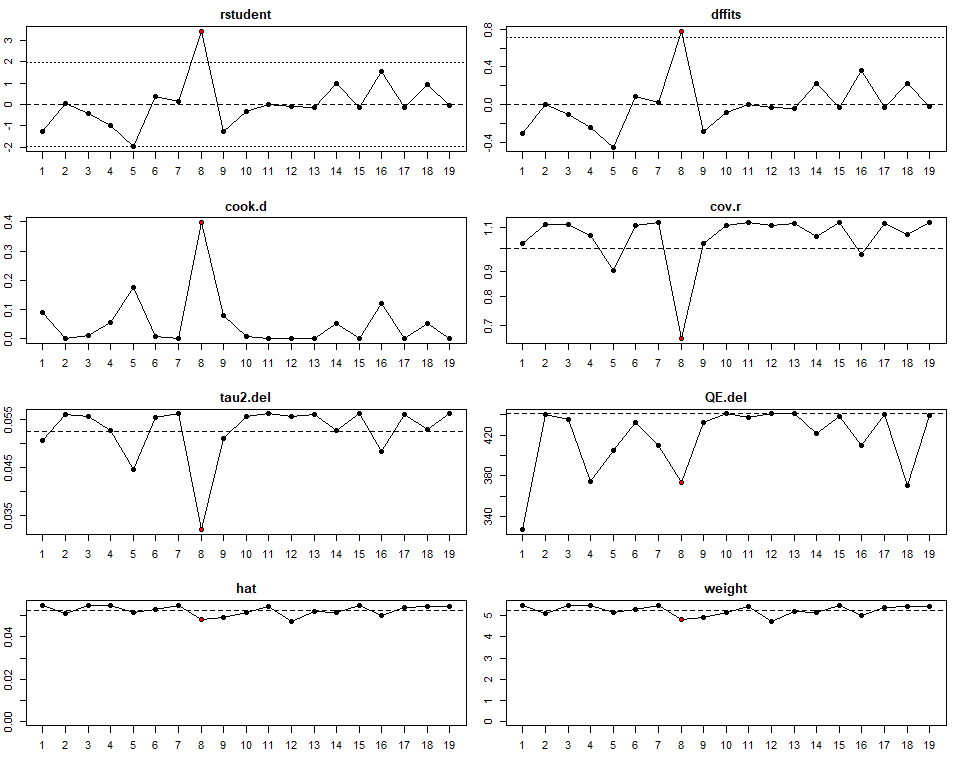

Supplement: Supplementary file 1 — Supplementary file1 (DOCX 2203 KB) [file 10143_2021_1619_MOESM1_ESM.docx]
